# Head and neck cancer: causes, prevention and treatment

**DOI:** 10.5935/1808-8694.20130041

**Published:** 2015-11-02

**Authors:** Ana Lívia Silva Galbiatti, João Armando Padovani-Junior, José Victor Maníglia, Cléa Dometilde Soares Rodrigues, Érika Cristina Pavarino, Eny Maria Goloni-Bertollo

**Affiliations:** a MSc in Health Sciences from FAMERP - São José do Rio Preto Medical School. Research Unit for Molecular Biology and Genetcs (UPGEM) (Biologist and Pharmacist; Doctoral student); b MD. PhD. (Adjunct Professor - São José do Rio Preto Medical School (FAMERP); c - MD. Associate Professor (Adjunct Professor in the São José do Rio Preto Medical School (FAMERP); d Nurse, PhD (Adjunct Professor - São José do Rio Preto Medical School (FAMERP); e Biologist, Associate Professor of Human Genetcs (Adjunct Professor - São José do Rio Preto Medical School (FAMERP). São José do Rio Preto Medical School (FAMERP)

**Keywords:** causality, disease prevention, head and neck neoplasms, primary treatment

## Abstract

**Abstract:**

Although head and neck carcinoma ranks fifth among cancer types, patient survival rates have not changed significantly over the past years.

**Objective:**

To determine the risk factors, causes, therapies, and prevention measures for head and neck cancer.

**Method:**

Risk factors, causes, therapies, and preventive measures for this disease were searched on databases PUBMED, MEDLINE, and SciELO.

**Results:**

Alcohol and tobacco are still atop risk factors. Other factors may influence the development of head and neck carcinoma. Surgery is the main treatment option, and the addition of radiotherapy following surgery is frequent for patients in the early stages of the disease. Other therapies target specific genetic molecular components connected to tumor development. Disease preventive measures include smoking cessation, limiting alcohol intake, preventing exposure to tobacco smoke and environmental carcinogenic agents, early detection of infection by HPV, maintaining oral health, good eating habits, and managing stress.

**Conclusion:**

Additional research is needed for a more thorough understanding of the development of head and neck carcinomas and to shed light on new ways to improve therapeutic approaches and interventions.

## INTRODUCTION

Head and neck squamous cell carcinoma (HNSCC) that comprising upper aerodigestive tract anatomic sites represents the third common cause of cancer death in worldwide. For the year 2009, approximately 47,000 HNSCC cases were estimated and 11,000 deaths from the disease were expected^1^.

The vast majority (more than 90%) are squamous cell carcinomas, and the disease typically appears in the oropharynx, oral cavity, hypopha-rynx, or larynx. The development of HNSCC is the result of the interaction of both environmental factors and genetic inheritance, and is therefore, multifactorial. Smoking and alcohol abuse are major risk factors for the development of this disease^2^.

Human papillomavirus (HPV) is also considered risk factor in about 25% of the disease. HPV infection has a known role in oropharyngeal carcinogenesis, particularly in tonsillar cancer, with strong and independent prognostic, probably because they determine the molecular profile of the cancer and thus the response to therapy. At the same time, not all smokers and alcohol users develop HNSCC, suggesting that individual variation in genetic susceptibility plays a critical role^3^. Preliminary results suggest that high-risk HPV infections seem to be biologically relevant in laryngeal carcinogenesis, however, the clinical significance of these infections and the implications in the disease prevention and treatment are unclear and require further investigation^4^.

The 5-year life expectancy is about 50% when there are lymphnode metastases^5^. It was demonstrate previously that cure rates in patients with advanced disease using tumor response to neoadjuvant chemotherapy is efficient. This is important because of the treatment intensity in future protocols so as to achieve the best cure rates with the least toxicity^6^. Nowadays, there are new surgical techniques, such as through robotic surgery, that decreases the tracheotomy rate, and allows a faster oral swallowing recovery and shorter hospital stay^7^. The radiotherapy and concomitant chemotherapy also had been demonstrate better survival rates for laryngeal preservation and locoregional con-trol^8^. Mortality and morbidity associated with these malignancies remains high, causing an impact on the quality of life and also in the treatment cost of these patients^9^. The head and neck cancer disease can affect overall and mental health, appearance, employment, social life and family living. Also may occur serious changes in the functioning of the upper aero digestive tract that affect the life quality of patients Furthermore, the understanding of disease development and its appearance can help in the treatment choice, as well as the symptoms analysis and/or rehabilitation necessary, better organization and quality of care, identifying aspects of impact on patient survival in help of the decision on the effectiveness of treatment through the clarification of the side effects of treatment. Based on the above data, this review focuses on recent advances related to causes, prevention, treatment, clinical aspects and outcomes in HNSCC.

## OBJECTIVE

To determine risk factors, causes, treatment and prevention of head and neck cancer through of the research in database (PUBMED, SciELO, MEDLINE).

## METHOD

We researched all papers published in the literature, regardless of year of publication, with following key words: “Head and neck cancer”, “Head and neck cancer and prevention”, “Head and neck cancer and causes”, “Head and neck cancer and treatment”, “Head and neck cancer and survival life”, “Head and neck cancer and tobacco”, “Head and neck cancer and alcohol”. In this study, we researched only papers that evaluated malignancies located in the upper aero digestive tract (oral cavity, pharynx and larynx cancer).

### Head and neck cancer causes

Tobacco smoking is well established as a dominant risk factor for HNSCC, and this risk is correlated with the intensity and duration of smoking habit^4^, ^10^. The cigarette contains nitrosamines and polycyclic hydrocarbons carcinogens elements that have genotoxic effects and therefore may increase the risk of disease. These elements can change the molecular profile of the individuals and cause mutations.

The study of Kumar et al.^11^ showed that smoking cessation reduces but does not eliminate the risk of cancer development, However, Marron et al.^12^ confirmed that cessation of tobacco smoking protect against the HNSCC development. The major risk factor for oral cancers among non-drinkers is tobacco use and among nons-mokers is alcohol use^13^, ^14^. The risk may increase directly with alcohol concentration (eg, consumption of spirits vs beer or wine), even after adjustment for total alcohol consumed. It is currently unclear whether the type of alcohol used affects the oral cancer risk after adjustment for total amount consumed and alcohol concentration^14^, ^15^.

Alcohol acts as a solvent to enhance mucosal exposure to carcinogens, increasing cellular uptake of these. The acetaldehyde, a metabolite of alcohol, can form DNA adducts, that interfere with DNA synthesis and repair^16^. According Marur & Forastiere^17^, the consumption of tobacco associated with alcohol consumption increases the HNSCC risk 40-fold. At the same time, not all smokers and alcohol users develop HNSCC, suggesting that individual variation in the genetic susceptibility plays a critical role^3^. Furthermore, there is a strong relationship between alcohol and tobacco use and the combined use of these further increases the risk^14^.

Recent data confirms that infection with HPV-16 is an independent risk factor for HNSCC, mainly for oropharyngeal squamous cell carcinoma^18^. In addition, high-risk HPV types (HR HPV) are a risk factors in about 25% of HNSCC, independent of other known risk factors, such as alcohol and tobacco^10^. Although the mode of transmission of HPV in head and neck cancer has not been determined, sexual behavior has been associated with an increased risk^19^. Moreover, the presence of viral DNA in tumors, the etiological link between HPV and head and neck cancer has been supported by the detection of HPV DNA in oral rinses and HPV-specific antibodies in head and neck cancer cases^19^.

The diet can be associated with decreased risk for the disease. Diet has strong evidence with cancer development and data confirm a probable causal relationship for a decreased HNSCC risk with non-starchy vegetables, fruits, and food containing carotenoids. A recent study confirmed that higher dietary pattern scores, with high consumption of fruit and vegetable and low intake of red meat, were associated with HNSCC reduced risk^20^.

Carcinogen exposure, oral hygiene, dental plaque formation, chronic irritation to the lining of the mouth, family history, low body mass index and exposure to ultraviolet light also all play a role, individually or in combination, in the HNSCC development, because they can modulate toxin and carcinogenic metabolism^21^, ^22^, ^23^.The carcinogen exposure increases the HNSCC risk because the carcinogens smoke has genotoxic effects. The cigarette has approximately 4,700 substances, and at least 50 of these are carcinogenic, including nitrosamines and polycyclic hydrocarbons^24^. Regarding oral hygiene, the polymicrobial supragingival plaque may be considered as a possible independent factor because it has a relevant mutagenic interaction with saliva, and individual oral health may be a co-factor in the development of oral cavity carcinomas. Perio-dontal diseases resulting from poor oral hygiene can lead to infections with consequent release of inflammatory mediators such as cytokines and the reactions against inflammation can promote cancer development. The loss of teeth can also contribute for oral cancer development, it leads for alteration of oral flora favors the reduction of nitrites and nitrates and the production of acetaldehyde, which leads to the formation of DNA adducts^21^, ^22^, ^23^, ^24^.

The influence of family history in HNSCC development may be because familial aggregations that may indicate that inheritable genetic factors play a role in HNSCC risk^22^. Several genetic polymorphisms in genes involved in the carcinogens metabolism, DNA repair or in several other processes have been associated with HNSCC risk, although the results were not always consistent. Since the differential ability to metabolize carcinogens happens only when exposure occurs, it is also possible that the familial risk reflects both a higher genetic susceptibility for HNSCC together with an aggregation of exposures^22^.

In a pooled analysis of 17 international studies, it was found that lean subjects were at higher risk for HNSCC, whereas heavy subjects were at a lower risk, compared with subjects with a normal body size, after adjustment for major HNSCC risk factors (smoking and drinking). One possible explanation is that, in the time shortly before diagnosis, undiagnosed cancer lesions in the head and neck may cause dysphagia or odynophagia or may alter taste and appetite, leading to a reduction of overall caloric intake and weight loss. The reduced risk among overweight people may indicate body size is a modifier of the risk associated with smoking and drinking. Further clarification may be provided by analyses of prospective cohort^23^.

Occupational activity also appears to be associated with HNSCC development. The study by Conway et al.^25^ showed that manual occupational activities, low income, low occupational-social class, low educational attainment and unemployment correlate with increased risk for disease development. The individuals who work in rural activities are in constant exposure to sunlight and in contact with carcinogenic substances that contribute to the development of oral cavity cancer^26^.

### Head and neck cancer treatment

The use of surgery, radiation, and/or chemotherapy depends on tumor respectability and location, as well as whether an organ preservation approach is feasible.^27^ The main treatment option for primary and secondary malignancy as well as recurrent disease is surgical therapy^28^. The use of transoral laser assisted surgery followed by radiotherapy is a common practice in the treatment of early stage oropharyngeal, hypopharyngeal and supraglottic carcinomas^29^. On the other hand early glottic carcinomas show excellent oncologic results after single modality treatment. Transoral laser surgery is the treatment of choice but radiotherapy is also a good alternative^30^.

Although obtaining negative surgical margins is the primary goal of head and neck surgery, achieving this may be impossible in some cases because of infiltration of vital structures such as the carotid artery or the prevertebral fasciae. The positive surgical margin status is associated with decreased survival, therefore a patient should be re-operated if the tumor was not removed completely^31^. However, achieving negative margins can cause impairment in important functions such as chewing, swallowing and speech, and adversely affect quality of life^32^. Therefore primary radiochemotherapy is an alternative for patients with advanced head and neck carcinomas.

Recommendation of planned neck dissection regardless of clinical response is supported by the high rates of residual disease observed in planned neck dissection surgical specimens and the data shows improved regional control and survival with planned neck dissection^33^. Advances in imaging techniques may help identify those patients with a clinical partial response for whom a planned neck dissection can be omitted. Until then, we recommend that patients achieving less than a clinical partial response after chemoradiation proceed to planned neck dissection^34^.

In general, there are 3 main approaches to the initial treatment of locally advanced disease: (1) concurrent platinum-based chemoradiation, with surgery reserved for residual disease; (2) surgery with neck dissection and reconstruction, followed by adjuvant radiation or chemoradiation, depending on the presence of adverse risk factors; or (3) induction chemotherapy followed by definitive chemoradiation and/or surgery Approximately 60% of patients with HNSCC present at a locally advanced stage, in which combined modality therapy with curative intent is recommended^27^, ^35^.

Cisplatin remains the cornerstone of treatment in recurrent and metastatic HNSCC. Moreover, postoperative concurrent administration of high-dose cisplatin with radiotherapy is more efficacious than radiotherapy alone in patients with locally advanced HNSCC and does not cause an undue number of late complications^36^. Data shows that radiation therapy combined with simultaneous 5-fluorouracil (5-FU), cisplatin, carboplatin, and mitomycin C as single drug or combinations of 5-FU with one of the other drugs results in a large survival advantage irrespective the employed radiation schedule. If radiation therapy is used as single modality, hyper-fractionation leads to a significant improvement of overall survival. Accelerated radiation therapy alone, especially when given as split course radiation schedule or extremely accelerated treatments with decreased total dose, does not increase overall survival^37^.

Cetuximab in combination with platinum/5-FU has emerged as a new alternative regimen for untreated patients based on results from the first-line Treatment of Recurrent or Metastatic Head and Neck Cancer trial. Cetuximab can be used with chemotherapy in first-line treatment of recurrent or metastatic disease, and in second-line treatment of platinum-refractory disease^35^. The data from a phase III trial support the role of cetuximab plus radiotherapy as an effective treatment option for patients with locoregionally advanced HNSCC. Moreover, cetuximab plus radiotherapy led to significant improvements in locoregional control and survival and these survival improvements may be maintained long-term, with a nine percentage point advantage for cetuximab plus radiotherapy in the 5-year overall survival rate, compared with radiotherapy alone. The combination of cetuximab and concurrent chemoradiotherapy is currently being investigated in phase III trials. Incorporation of cetuximab into sequential chemotherapy and radiotherapy/chemoradiotherapy regimens is yielding interesting results. After induction chemotherapy, the combination of cetuximab and radiotherapy was better tolerated than platinum-based concurrent chemoradiotherapy with a similar short-term rate of larynx preservation^38^.

The taxanes docetaxel and paclitaxel (Taxol®) are active in HNSCC. Several phase II studies have indicated that adding a taxane improves responsiveness to 5-FU based induction chemotherapy. Results of a randomized phase III trial that compared induction chemotherapy using docetaxel and 5-FU together with 5-FU alone indicated that incorporation of a taxane substantially improves clinical response and survival in locally advanced head and neck cancer. However, paclitaxel may be develop neurotoxicity and be problematic, particularly when used in combination with other neurotoxic agents such as cisplatin^39^.

Regarding to radiotherapy (RT), RT intensity modulated (IMRT) has increasingly been shown to be advantageous compared with traditional techniques such as conventional RT (2D) and conformation (3D), in that it provides a more homogeneous coverage of dose to the target volume and a decrease in the dose in the surrounding tissues. The highest dose is related with better tumor control and better survival rates^40^.

There is also hyperfractionated radiation therapy utilized in patients with HNSCC. However, this treatment option can develop reaction of different intensities in the mucosa, as oral mucositis, that causes significant pain, chewing and swallowing difficulties and is considered the most debilitating acute reaction during head and neck cancer treatmen^41^. The use of brachytherapy treatment in patients with HNSCC, that use radiation sources in direct contact with the tissues to be irradiated, increase the risk to develop soft tissue necrosis, which may be defined as an ulcer located in the radiated tissue, without the presence of residual malignancy^42^.

Actually, there are novel therapies that target specific molecular components that can improve understanding of the molecular genetic for HNSCC. For example, the EGFR (Epidermal growth factor receptor), that overexpress more than 90% in the HNSCC, a central transducer of multiple signaling pathways is involved in tumor cell growth, angiogenesis, and invasion. There are different points along this signal transduction sequence and the therapy can target in an effort to blockade EGFR function. If this blockade occur in combination with other treatment modalities, for example the inhibition of other signaling pathways, the EGFR blockade may be most successful^11^.

The availability of biologic therapies that target mechanisms important in tumor growth and metastasis has led to efforts to personalize therapy based on specific patient or tumor characteristics. Studies have included either subgroup or correlative analyses of such characteristics with outcome^35^, ^43^. Interdisciplinary collaboration and case discussions should take place in the context of a tumor board. Further progress may be expected as new insights are obtained about key mechanisms and prognostic factors involved in HNSCC.

### Head and neck cancer prevention

New approaches are helping to elucidate long-recognized but poorly understood biologic concepts such as field cancerization and are helping to explain perplexing clinical patterns such as local tumor recurrence following seemingly complete resection^44^. Analysis of the molecular genetic changes in the HNSCC discloses not just individual tumor differences, but also consistent large-scale differences that permit the recognition of important subtypes of HNSCC. The novel treatment strategies can be improve these differences that to enhance immunologic responses to tumor-specific antigens and to target individual components of the molecular genetic apparatus^45^.

A number of definitive risk factors such as smoking, HPV infection and key genetic alterations including EGFR, TP53, p16, p14 were identified and many will be discovered in the coming years. In the current context, by quitting cigarette smoking, limiting alcohol drinking, avoiding tobacco chewing, preventing exposure to second hand tobacco smoke, environmental carcinogens, screening for HPV, maintaining good oral health, nutritional habits and managing stress could be good primary measures for preventing or delaying HNSCC development^46^. However, data are discontented and demonstrated that smoking and etilism maintenance and/or recurrence rates are high in patients treated for HNSCC, meaning only patient advice is not enough as a strategy leading to these habit cessation^47^.

The poor prognosis for HNSCC is primarily due to disease detection at advanced stages. Therefore, the understanding of the field cancerization and the molecular genetics of HNSCC is essential to provide better intervention and therapeutic approaches, thus introducing various biomarkers with potential application for diagnosing, staging, monitoring, and prognosticating^2^.

The diet can also influence in neoplasias development due to the way in which they are prepared and the additives used. Certain foods having antitumoral properties, such cruciferous plants (cauliflower, broccoli and cabbage), they blocked enzymes responsible for tumoral activation or chelation (sequestering) of the free radicals, an enhanced detoxification process that alters the activity of these enzymes or else the modulation of certain DNA repair processes^48^. Therefore, a regular diet may prevent or delay the HNSCC development.

Although there is data showing instruments for preventing head and neck cancer, it is necessary for further clarification more studies for detection of the new prognostic indicators, which could be used in diagnostics.

## RESULTS

Alcohol and smoking remain the major risk factors and have an additive effect. However, there are other factors that also influence the HNSCC development as HPV infection, diet, carcinogen exposure, oral hygiene, infectious agents, family history, low body mass index, exposure to ultraviolet light, chronic irritation to the lining of the mouth and dental plaque formation, preexisting medical conditions and occupational activity.

The main treatment option for primary and secondary malignancy as well as recurrent disease is surgical therapy and the use of surgical practice followed by radiotherapy is a common practice in the treatment for HNSCC in early stages of the disease (I or II) with a high percentage of cure. There are also novel therapies that target specific components of the molecular genetic apparatus supporting tumor development and growth.

The quitting cigarette smoking, limiting alcohol drinking, avoiding tobacco chewing, preventing exposure to second hand tobacco smoke, environmental carcinogens, screening for HPV, maintaining good oral health, nutritional habits and managing stress could be good primary measures for preventing or delaying HNSCC development.

## DISCUSSION

The HNSCC is the fifth leading cause of death in the world population, with an incidence of 500,000 new cases per year. In recent years, the management of head and neck cancer has been more complex with combined-modality programs, as well as the integration of new diagnostic and therapeutic technologies^20^.

Nowadays, it is known that is not only smokers and drinkers that may develop HNSCC, although they still have a great influence. There are many other risk factors that are involved in the HNSCC appearance, for this reason it is necessary to investigate the origin of the disease in each patient. Thereby, the choice of treatment may be more specific. HNSCC is the most complex “organ site”, so the treatment decision is not an overstatement, and supports a best practices model of multidisciplinary team involvement. Surgery is often required, followed by treatment of a radiotherapy or chemotherapy.

Surgery may be disfiguring and psychologically traumatic, however, there are methods that can preserve the organs, such as preservation of mandibule, because the mandibule has important roles in functional, aesthetic, psychological aspects of the human. The conservative mandibulectomy techniques presents favorable results^49^. There are another rehabilitation methods, as rehabilitation methods reconstruction of all cervicofacial post-excision defects and reconstruction using osteomyocutanous grafts and microanastomosis^50^. Although there surgery options for organ preservation, these methods still have limitation and needs more investigations.

Chemotherapy, either induction therapy or concurrent chemoradiotherapy, is routinely integrated into the treatment of patients with locally advanced head and neck cancer. Sequential therapy incorporating both induction chemotherapy and chemoradiation is a feasible approach and has the potential to further improve survival outcomes^39^. However, higher doses of the chemotherapies or radiotherapy can be lead to many collateral effects that can be prejudicial and affect the life quality of patients.

Nowadays, the most effective measures to improve the prognostic of the malignant tumors are prevention and early diagnosis. The early detection and initial treatment are successfully treated when HNSCC is discovered. If not detected early may require treatments ranging from surgery for its removal to radiotherapy or chemotherapy. The main problem is that disinformation and non-compliance of the symptoms by the patients, and lack of routine examinations by health professionals are causes of late diagnosis of tumor. Therefore, this leads to a stronger treatment that can impair life quality of the patient due to HNSCC as one of the most aggressive and mutilating tumor.

## CONCLUSION

Although there are established risk factors, it is known that there are many other factors that may contribute to HNSCC development. There are different types of treatment for HNSCC according to the disease stage. Some have already been confirmed to increase survival of patients, however, all types of treatment (surgery, chemotherapy, radiotherapy and chemoradiotherapy) lead to side effects that may impair the patient's life and mutilation of certain organs. New insights for intervention and therapeutic approaches are needed to more complete understanding of HNSCC development.
